# Supplementary material for: A Meta-Analysis of Group Cognitive Behavioral Therapy and Group Psychoeducation for Treating Symptoms and Preventing Relapse in People Living with Bipolar Disorder
Source: Healthcare (Basel). 2022 Nov 15;10(11):2288. doi: 10.3390/healthcare10112288 (PMC9691241; doi:10.3390/healthcare10112288)
Supplement: Supplementary file 1 [file healthcare-10-02288-s001.zip › Supplementary Figure S1_ Risk of Bias Graph (1).pdf]

	Random sequence generation (selection bias)	Deviations from Intended interventions	Missing Outcome Data	Selective reporting (reporting bias)	Measurement of Outcome	Overall Risk of Bias
Candini. 2013	−	−	+	+	+	?
Castle 2010	+	?	+	+	+	?
Chen 2018	+	?	+	+	?	?
Colom 2003	+	?	+	?	+	?
Costa 2012	+	?	+	?	?	?
Gomes 2011	+	+	+	?	+	?
Kallestad. 2016	+	+	+	+	?	?
Kessing 2018	+	+	+	+	+	+
Lin 2020	+	?	+	?	+	?
Morris 2016	+	+	+	?	+	?
Russel 2010	+	+	+	?	+	?

Figure S1: Risk of Bias Graph for Included Studies
