# Supplementary material for: A Meta-Analysis of Group Cognitive Behavioral Therapy and Group Psychoeducation for Treating Symptoms and Preventing Relapse in People Living with Bipolar Disorder
Source: Healthcare (Basel). 2022 Nov 15;10(11):2288. doi: 10.3390/healthcare10112288 (PMC9691241; doi:10.3390/healthcare10112288)
Supplement: Supplementary file 1 [file healthcare-10-02288-s001.zip › Supplementary Figure S2a, b, c_ Funnel Plots (1).pdf]

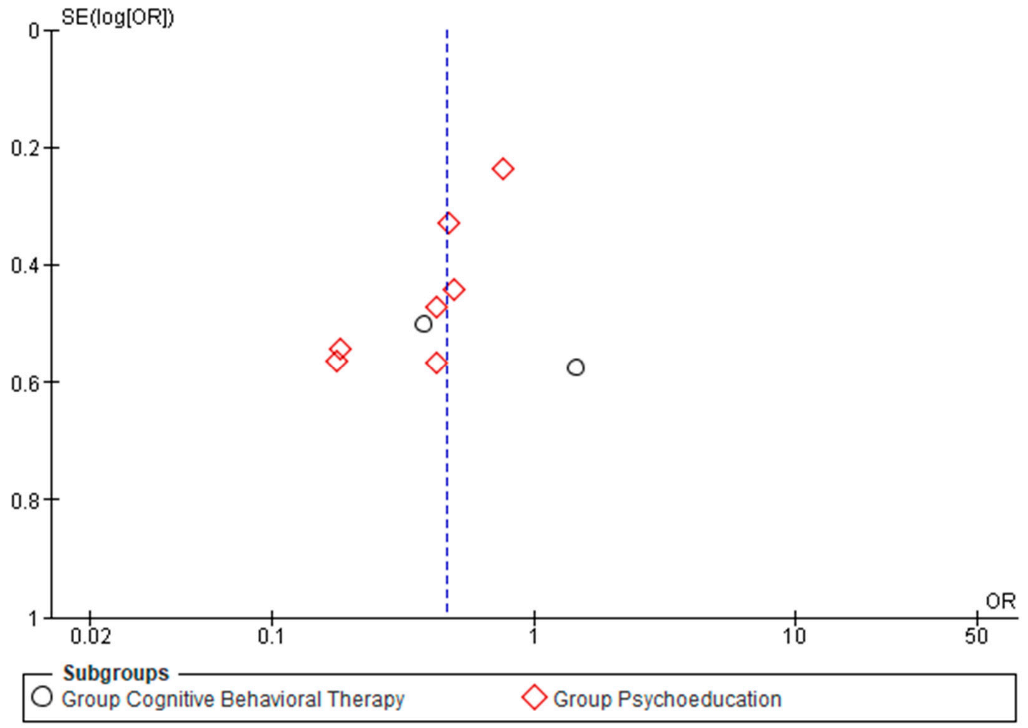

Figure 2a: Funnel Plot for Studies that investigate relapse rates as an outcome

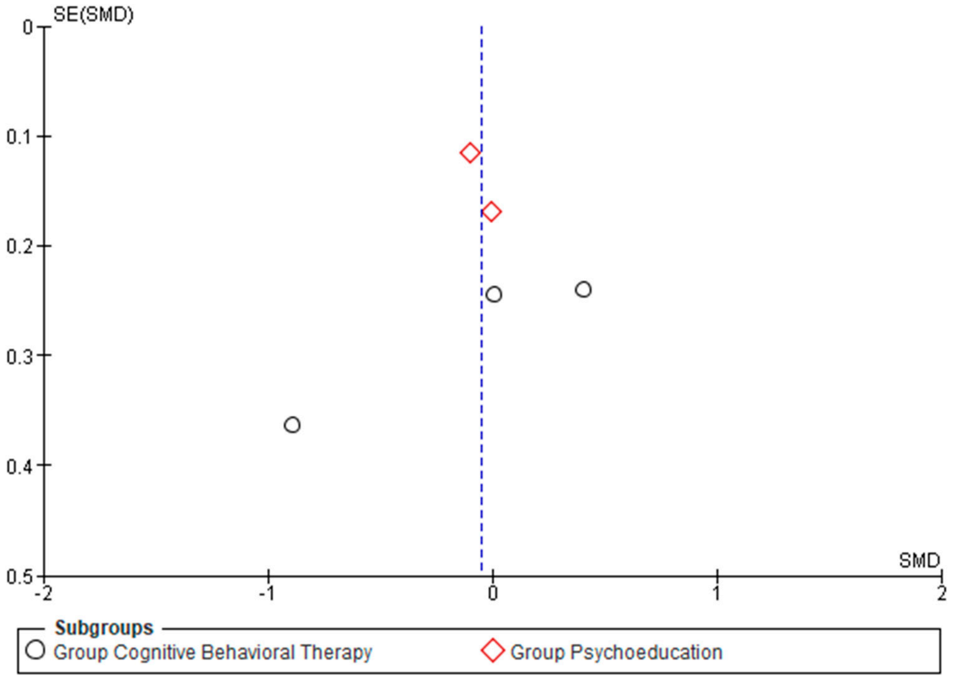

Figure 2b: Funnel Plot for Studies that investigate depressive symptoms as an outcome

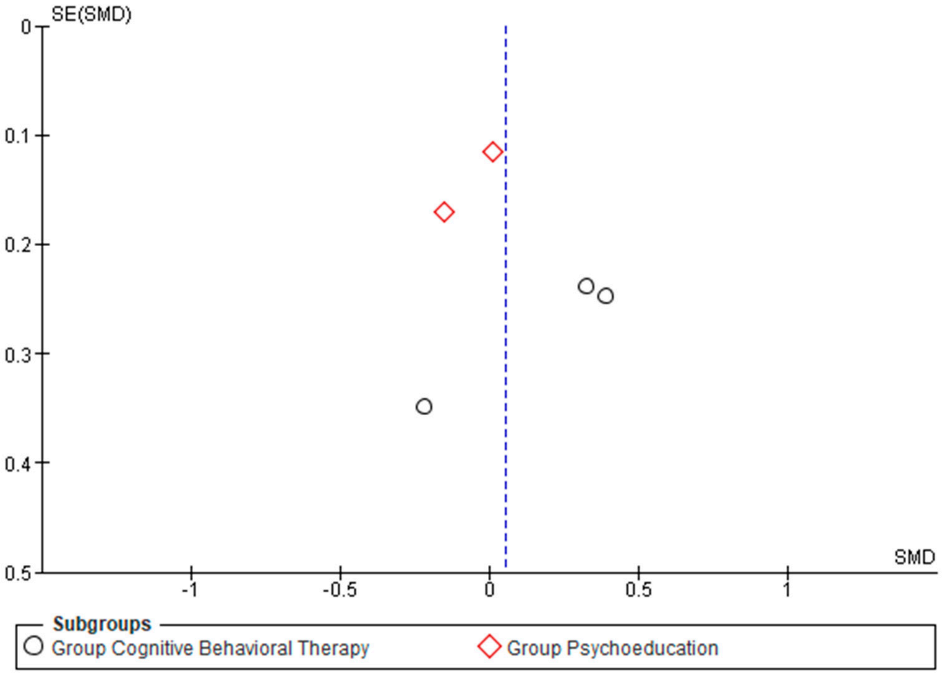

Figure 2c: Funnel Plot for Studies that investigate manic symptoms as an outcome
