# Supplementary material for: A Meta-Analysis of Group Cognitive Behavioral Therapy and Group Psychoeducation for Treating Symptoms and Preventing Relapse in People Living with Bipolar Disorder
Source: Healthcare (Basel). 2022 Nov 15;10(11):2288. doi: 10.3390/healthcare10112288 (PMC9691241; doi:10.3390/healthcare10112288)
Supplement: Supplementary file 1 [file healthcare-10-02288-s001.zip › Supplementary Table S1_ Search Strategy_Updated.pdf]

Supplemental Table S1. PubMed Search Strategy:

GCBT

Search Number	Search Terms
#1	(Bipolar disorder OR bipolar OR manic-depressive psychosis OR bipolar affective disorder OR bipolar depression)
#2	(Group Cognitive therapy OR Group behavioral therapy OR Group Cognitive Behavioral Therapy OR Group CBT OR Group Psychotherapy)
#3	#1 and #2
#4	#3 AND “Clinical Trial” [Publication Type]

GPE

Search Number	Search Terms
#1	(Bipolar disorder OR bipolar OR manic-depressive psychosis OR bipolar affective disorder OR bipolar depression)
#2	(Group Psychoeducation OR Group Education)
#3	#1 and #2
#4	#3 AND “Clinical Trial” [Publication Type]

PsychINFO Search Strategy

GCBT

Search Number	Search Terms
#1	(Bipolar disorder OR bipolar OR manic-depressive psychosis OR bipolar affective disorder OR bipolar depression)
#2	(Group Cognitive therapy OR Group behavioral therapy OR Group Cognitive Behavioral Therapy OR Group CBT OR Group Psychotherapy)
#3	#1 and #2
#4	#3 AND (Clinical Trial)

GPE

Search Number	Search Terms
#1	(Bipolar disorder OR bipolar OR manic-depressive psychosis OR bipolar affective disorder OR bipolar depression)

#2	(Group Psychoeducation OR Group Education)
#3	#1 and #2
#4	#3 AND (Clinical Trial)

Embase Search Strategy

GCBT

Search Number	Search Terms
#1	(Bipolar disorder OR bipolar OR manic-depressive psychosis OR bipolar affective disorder OR bipolar depression)
#2	(Group Cognitive therapy OR Group behavioral therapy OR Group Cognitive Behavioral Therapy OR Group CBT OR Group Psychotherapy)
#3	#1 and #2
#4	#3 AND ([controlled clinical trial]/lim OR [randomized controlled trial]/lim)

GPE

Search Number	Search Terms
#1	(Bipolar disorder OR bipolar OR manic-depressive psychosis OR bipolar affective disorder OR bipolar depression)
#2	(Group Psychoeducation OR Group Education)
#3	#1 and #2
#4	#3 AND ([controlled clinical trial]/lim OR [randomized controlled trial]/lim)

CENTRAL Search Strategy

GCBT

Search Number	Search Terms
#1	(Bipolar disorder OR bipolar OR manic-depressive psychosis OR bipolar affective disorder OR bipolar depression)
#2	(Group Cognitive therapy OR Group behavioral therapy OR Group Cognitive Behavioral Therapy OR Group CBT OR Group Psychotherapy)
#3	#1 and #2
#4	#3 AND (Clinical Trial)

GPE

Search Number	Search Terms
#1	(Bipolar disorder OR bipolar OR manic-depressive psychosis OR bipolar affective disorder OR bipolar depression)
#2	(Group Psychoeducation OR Group Education)
#3	#1 and #2
#4	#3 AND (Clinical Trial)

*GCBT: Group Cognitive Behavioral Therapy
**GPE: Group Psychoeducation
