# Supplementary material for: A Meta-Analysis of Group Cognitive Behavioral Therapy and Group Psychoeducation for Treating Symptoms and Preventing Relapse in People Living with Bipolar Disorder
Source: Healthcare (Basel). 2022 Nov 15;10(11):2288. doi: 10.3390/healthcare10112288 (PMC9691241; doi:10.3390/healthcare10112288)
Supplement: Supplementary file 1 [file healthcare-10-02288-s001.zip › Supplementary Table S2_Characteristics of Excluded Studies.pdf]

Supplementary Table S2. Characteristics of Excluded Studies

Study	Reasons for exclusion
Camacho E.M., Ntais D., Jones S., Riste L., Morriss R., Lobban F, et al. Cost-effectiveness of structured group psychoeducation versus unstructured group support for bipolar disorder: Results from a multi-centre pragmatic randomized controlled trial.. Journal of Affective Disorders Mar 2017;211:27-36.	Ineligible Outcome Measure
T.Y. Gulsum, Guriz S.O., K.K. Akfer, K. Ahmet, O. Sibel. Effectiveness of the group psychoeducation program in bipolar disorder. Journal of Cognitive Behavioral Psychotherapy and Research 2020;9(2):73-81.	Non-English
Hubbard, A.A., McEvoy, P.M., Smith, L., Kane, R.T.. Brief group psychoeducation for caregivers of individuals with bipolar disorder: A randomized controlled trial.. Journal of Affective Disorders Aug 2016;200:31-36.	Ineligible Population
V. Kazempour, H. Ebrahimi, M.A. Jafarabadi, S.G. Nourazar, H. Zamani. The Effect of Group Cognitive Behavioral Therapy on Cognitive Emotion Regulation Strategies of Adolescents with Bipolar Disorder During Their Euthymic Phase: A Randomized, Controlled Trial. Iranian Red Crescent Medical Journal May 2018.	Ineligible Population
Kopsahili I., Papadaki R., Giakoumaki S.G., Tsapakis E.M.. Effectiveness of group cognitive behavioral therapy (G-CBT) in bipolar affective disorder patients. In: Europeon Psychiatry. Vol. 28. 2013.	Ineligible Study Design
W. Luisa, L.C.C. Muriel, B. Gilles, Weibel S.. Patients' Perspective of the Impacts of Group Psychoeducation for Bipolar Disorder: A Qualitative Study. The Journal of Nervous and Mental Disease Jan 2022;210(1):71-76.	Ineligible Outcome Measure
Madigan K., Egan P., Brennan D., Hill S., Maguire B., Horgan F. et al. A randomised controlled trial of carer-focussed multi-family group psychoeducation in bipolar disorder. European Psychiatry May 2012;27(4):281-284.	Ineligible Population
S.V. Parikh, A. Zaretsky, S. Beaulieu, L.N. Yatham, L.T. Young, I. Patelis-Siotis, et al. A	Non-Quantitative Data

Randomized Controlled Trial of Psychoeducation or Cognitive-Behavioral Therapy in Bipolar Disorder: A Canadian Network for Mood and Anxiety Treatments (CANMAT) Study. Journal of Clinical Psychiatry Jun 2012;73(6):803-10.	
Parkins, Monica M.. A randomized controlled trial of group cognitive-behavioral therapy for patients with bipolar disorder: Effects on social functioning and quality of life.. Dissertation Abstracts International 2015.	Ineligible Outcome Measure
I. Patelis-Siotis, L.T. Young, J.C. Robb, M. Marriott, P.J. Bieling, L.C. Coxm, et al. Group cognitive behavioral therapy for bipolar disorder: a feasibility and effectiveness study. Journal of Affective Disorders Jul 2001;65(2):145-53.	Ineligible Study Design
Prieto J.F.P., Gonzalez P.M., Vilaplana N.L.. Group psychoeducation for patients with bipolar disorder [Intervencion grupal psicoeducativa en el trastorno bipolar]. Anales de Psiquiatria Jul 208;24(4):159-167.	Non-English
Provencher M.D., Abry J., Mirabel-Sarron, C., Beaulieu, S.. Group psychoeducation and cognitive-behavioral therapy for bipolar disorder [La psychoeducation et la TCC de groupe pour le trouble bipolaire.]. Revue Francophone de Clinique Comportementale et Cognitive Nov 2016;21(2):38-57.	Non-English
A. Aretsky, W. Lancee, C. Miller, A. Harris, S.V. Parikh. Is Cognitive-Behavioural Therapy More Effective Than Psychoeducation in Bipolar Disorder? Canadian Journal of Psychiatry Jul 2008;53(7):441-8.	Non-Quantitative Data
Zyto S., Jabben N., Schutle P.F.J., Regeer, E.J., Goossens, P.J.J., Kupka, R.W.. A multi-center naturalistic study of a newly designed 12-sessions group psychoeducation program for patients with bipolar disorder and their caregivers. International Journal of Bipolar Disorders Sep 2020;8.	Ineligible Population
